# Evaluation of DNA ploidy in relation with established prognostic factors in patients with locally advanced (unresectable) or metastatic pancreatic adenocarcinoma: a retrospective analysis

**DOI:** 10.1186/1471-2407-9-264

**Published:** 2009-07-31

**Authors:** Nikolas Tsavaris, Nicolaos Kavantzas, Kostantinos Tsigritis, Ioannis D Xynos, Nikitas Papadoniou, Andreas Lazaris, Christos Kosmas, George Agrogiannis, Anna Dokou, Evangelos Felekouras, Efstathios Antoniou, Aris Polyzos, John Sarantonis, Heracles Tsipras, Gavrilos Karatzas, Alexandros Papalambros, Efstratios S Patsouris

**Affiliations:** 1 Oncology Unit, Department of Pathophysiology, Laikon General Hospital, Athens University School of Medicine, Athens, Greece; 2 1st Department of Pathology, Laikon General Hospital, Athens University School of Medicine, Athens, Greece; 3 1st Department of Surgery, Laikon General Hospital, Athens University School of Medicine, Athens, Greece; 4 2nd Department of Medical Oncology, Metaxa Cancer Hospital, Piraeus, Greece; 5 2nd Department of Surgery-Propedeutic, Laikon General Hospital, Athens University School of Medicine, Athens, Greece; 6 Oncology Unit, 1st Department of Internal Medicine – Propedeutic, Laikon General Hospital, Athens University School of Medicine, Athens, Greece; 7 3rd Department of Surgery, "G. Gennimatas" General Hospital, Athens, Greece; 8 3rd Department of Surgery, Attiko Hospital, University of Athens School of Medicine, Athens, Greece; 9 Department of Gastrenterology, "Andreas Papandreou" General Hospital, Rhodes, Greece

## Abstract

**Background:**

Most patients with ductal pancreatic adenocarcinoma are diagnosed with locally advanced (unresectable) or metastatic disease. The aim of this study was to evaluate the prognostic significance of DNA ploidy in relation with established clinical and laboratory variables in such patients.

**Methods:**

Two hundred and twenty six patients were studied retrospectively. Twenty two potential prognostic variables (demographics, clinical parameters, biochemical markers, treatment modality) were examined.

**Results:**

Mean survival time was 38.41 weeks (95% c.i.: 33.17–43.65), median survival 27.00 weeks (95% c.i.: 23.18–30.82). On multivariate analysis, 10 factors had an independent effect on survival: performance status, local extension of tumor, distant metastases, ploidy score, anemia under epoetin therapy, weight loss, pain, steatorrhoea, CEA, and palliative surgery and chemotherapy. Patients managed with palliative surgery and chemotherapy had 6.7 times lower probability of death in comparison with patients without any treatment. Patients with ploidy score > 3.6 had 5.0 times higher probability of death in comparison with patients with ploidy score < 2.2 and these with ploidy score 2.2–3.6 had 6.3 times higher probability of death in comparison with patients with ploidy score < 2.2.

**Conclusion:**

According to the significance of the examined factor, survival was improved mainly by the combination of surgery and chemotherapy, and the presence of low DNA ploidy score.

## Background

Carcinoma of the pancreas is a very aggressive tumor, posing the fourth leading cause of cancer-related death in the United States [1,2]. Most patients with pancreatic ductal adenocarcinoma present with locally advanced or metastatic disease on diagnosis, despite the availability of advanced imaging techniques. Only 10–20% of cases are candidates for curative surgery [3-5] and in these cases, the reported 5-year survival rate ranges between 11% and 25% [6-8] as persistence or recurrence of regional disease is reported in approximately 80% of patients following curative resection [9]. Subsequently, surgery for pancreatic cancer plays frequently a palliative role, to cure jaundice, obstruction or pain [10]. Surgical palliation on the other hand appears to be associated with a higher rate of early complications and possibly a higher rate of procedure related mortality [11], while stents may become obstructed causing recurrent jaundice [12].

To improve the prognosis of patients with pancreatic cancer, it is essential to provide non-surgical treatment options, such as systemic chemotherapy or targeted therapy [13]. Systemic chemotherapy for pancreatic cancer has proven of limited value because of the low response rates and the severe adverse effects. Patients suitable for chemotherapy should, therefore, be carefully selected on the basis of specific prognostic factors. Several studies have reported various pre- and postoperative factors as determinants of short- and long-term survival in patients undergoing surgery, but little is known about prognostic indices for survival in patients with unresectable disease [14-26].

The influence of DNA content on prognosis in adenocarcinomas of the pancreas has been investigated occasionally, and the results are controversial. The findings published in the literature, suggest that additional studies are required to obtain the prognostic impact of DNA content in pancreatic cancer [27].

In a previously published study by our group we have identified a number of factors which had independent impact on survival including tumor localization, metastases, PS, jaundice, weight loss, CRP, raised CEA and CA-19.9, palliative surgery and chemotherapy [28]. In the present study we update our patient cohort with the addition of a new laboratory parameter, DNA content.

## Methods

### Patients and data sources

The medical records of 226 patients between 1997 and 2003, with a histological diagnosis of pancreatic adenocarcinoma, from five Greek general hospitals were retrospectively reviewed. All had advanced unresectable pancreatic adenocarcinoma. For the diagnosis of distant metastasis, various imaging modalities were used, including chest X-ray, ultrasonography and computed tomography. Pathological confirmation of ductal adenocarcinoma was obtained by surgery or a fine-needle aspiration biopsy (FNAB). Survival time was calculated from time of diagnosis to death due to pancreatic cancer-related complications. Records with complete data (for the parameters used as prognostic factors) were included in the analysis. This protocol has been approved (ID:6443, 15/03/2004) by the National and Kapodistrian University of Athens human research ethics committee.

### Prognostic variables

Twenty-two possible prognostic variables were selected, based on factors identified by previous studies [6-8,14-26,28] (Table 1). Histopathological grading was based on the WHO system [29]. Patients were staged according to the International Union Against Cancer TNM classification [30].

**Table 1:** Demographic and clinical variables in the study population (n = 226)

Variable	Labels	n	%
Gender	Males	132	61.4
	Females	83	38.6
Age	≤ 60	87	40.5
	60+	128	59.5
PS	≥ 90	25	11.1
	= 80	75	33.2
	= 70	74	19.5
	= 60	40	17.7
	≤ 50	42	18.6
Location	Head	147	68.4
	Body	52	24.2
	Back ('tail")	16	7.4
Therapy	Chemotherapy and surgery	53	24.7
	Chemotherapy only	65	30.2
	Surgery only	24	11.2
	None	73	34.0
Surgery	Yes	77	35.8
	No	138	64.2
Chemotherapy	Yes	118	54.9
	No	97	45.1
Ploidy	< 2.2	20	8.8
	2.2–3.6	86	38.1
	>3.6	120	53.1
Amylase	Yes	23	10.7
	No	192	89.3
Grading	High	14	6.5
	Medium	187	87.0
	Low	14	6.5
Primary tumor extent	1	15	7.0
	2	60	27.9
	3	77	35.8
	4	63	29.3
CEA	≤ 5 mg/dL	72	33.5
	>5 mg/dL	143	66.5
CA 19-9	≤ 30 × normal	30	14.0
	>30 × normal	185	86.0
Thrombophlebitis	Yes	105	48.8
	No	110	51.2
Diabetes	Yes	199	92.6
	No	16	7.4
CRP	Normal	150	69.8
	Increased	35	16.3
	Greatly increased	30	14.0
Steatorrhoea	Yes	99	46.0
	No	116	54.0
Albumin	Yes	114	53.0
	No	101	47.0
Epoetin	Yes	36	16.7
	No	179	83.3
Transfusion	Yes	61	28.4
	No	154	71.6
Weight loss	None	62	28.8
	1–5%	31	14.4
	6–10%	58	27.0
	>10%	64	29.8
Pain	None	50	23.3
	Pain 1	84	39.1
	Pain 2	55	25.6
	Pain 3	26	12.1
Metastases	None	86	40.0
	LN	37	17.2
	LIV	7	3.3
	AB	29	13.5
	LN-AB	30	14.0
	LIV-LN-AB	26	12.1
Jaundice	Yes	46	21.4
	No	169	78.6

AB, abdominal metastasis; CA 19-9, cancer antigen 19-9; CEA, carcinoembryonic antigen; CRP, C-reactive protein; LIV, liver metastasis; LN, lymph node metastasis; 1: tumor < 2 cm confined to the panreas, 2: tumor > 2 cm confined to the pancreas, 3: invasion of adjacent tissues by tumor, 4: invasion of adjacent organs.

For the evaluation of continuous biochemical parameters we used group categorisations: for C-reactive protein (CRP) normal <5 mg/dL, elevated: 5–15 mg/dL, >15 mg/dL); for cancer antigen 19-9 (CA 19-9); patients with values ≤ 30 × nl vs >30 × nl; for carcinoembryonic antigen (CEA) normal ≤ 5 mg/dL and elevated >5 mg/dL; for amylase: yes and no; for hypoalbuminaemia: yes and no; for diabetes: yes and no. Anemia, before initiation of any therapy, was presented in two groups; severe (patients received blood transfusion), and moderate (patients received therapy with epoetin). For clinical parameters similar categorisations were used. For therapy: palliative surgery followed by chemotherapy, surgery only, chemotherapy only, supportive care only; for performance status (PS): ≥90, = 80, = 70, = 60 and ≤50; for jaundice: yes and no; for thrombophlebitis: yes and no; for steatorrhoea: yes and no; for weight loss: none, 1–5%, 6–10% and >10% of the total body weight. Pain was graded as follows: grade 0: absence of pain, grade 1: palliation with common analgesics (paracetamol, NSAIDs), grade 2: controlled with the use of opioids (fentanyl or morphine), grade 3: does not remit completely despite the use of opioid analgesics. For location of cancer: head, body and back (tail) of pancreas; for primary tumor extent: tumor < 2 cm confined to the pancreas, tumor > 2 cm confined to the pancreas, invasion of adjacent tissues by tumor, invasion of adjacent organs and for distant metastasis: yes and no. For ploidy score, group categorisation was also applied: <2.2, 2.2–3.6, >3.6.

### DNA Measurements (Ploidy)

**Figure 1:**
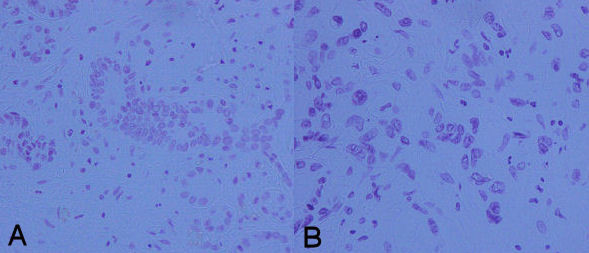
**DNA index 1.9 (A) versus DNA index 4.4 (B) as derived by subsequent image analysis of pancreatic tumor specimens (Feulgen staining, 200×)**.

**Figure 2:**
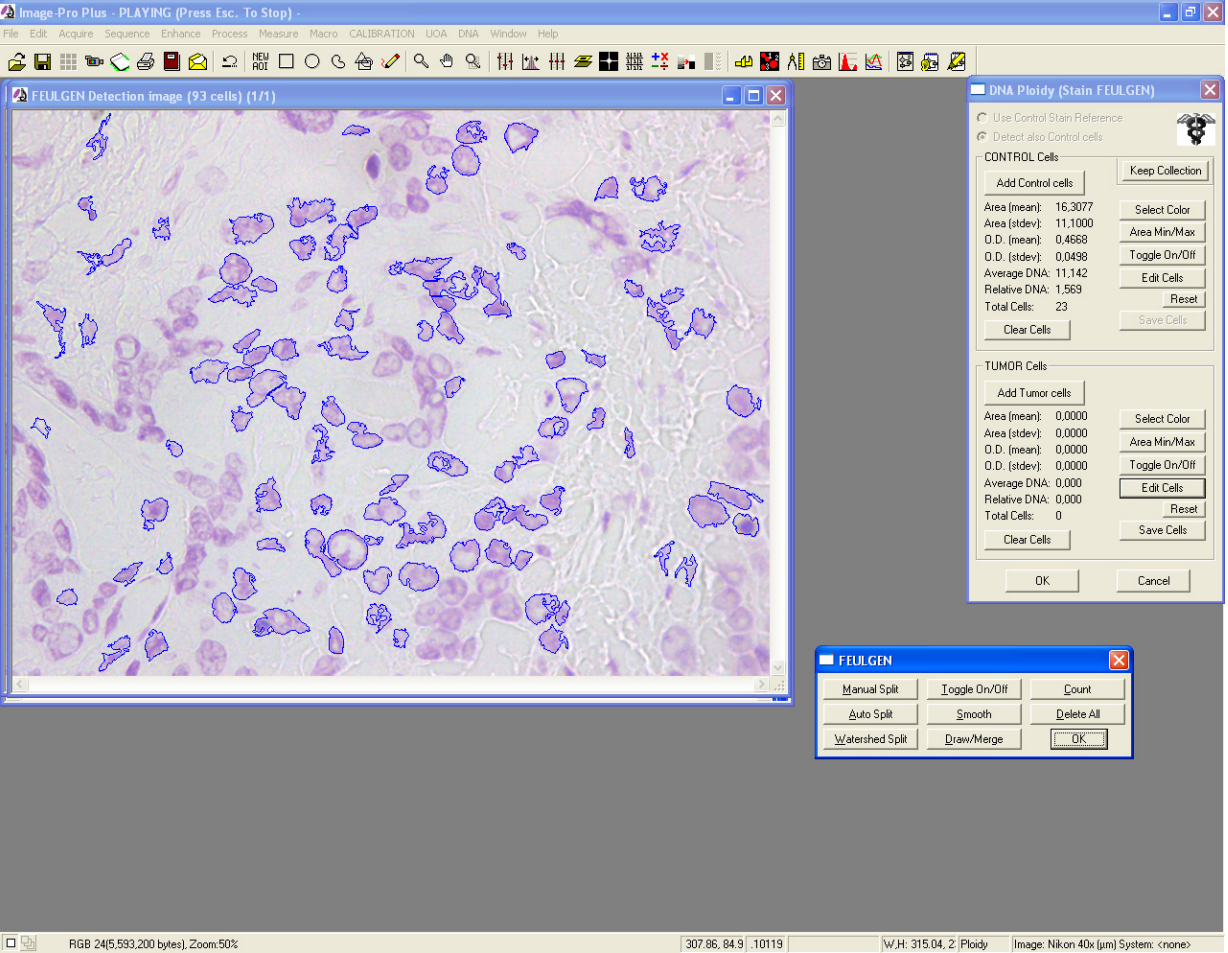
**Representative snapshot from image analysis screen (DNA ploidy)**. Nuclei are semiautomatically circumscribed and measured.

The nuclei of Feulgen-stained cells were evaluated for DNA ploidy using a Nikon eclipse microscope (Nikon, Japan) connected with a Nikon CCD videocamera and an IBM Pentium 4/PC with the appropriate Cell Measurement Software (Image Pro Plus v. 5.1, Media Cybernetics Inc, Silver Springs, MD, USA). A total of 100–200 nuclei with clear boundaries appearing to have no loss of membrane integrity were identified for analysis from each tissue sample. Measurements were made using a magnification of ×200. This analysis configuration permits operator-dependent selection and measurement of DNA content (Figures 1 and 2). This cell measurement system was calibrated before each analysis session using a slide with human normal lymphocytes with known DNA content. The data generated were downloaded to standard software packages for final analysis. DNA histograms were categorized as diploid if the histogram presented a single peak (2c; c = haploid DNA content) in the G0–G1 area and the cell nuclei population did not exceed 10% in the G2 region (4c). A sample was considered aneuploid if clear aneuploid peaks (3c, 5c, 7c and 9c) were present. For each case, coefficient of variance (CV) and DNA index/ploidy score was calculated relative to internal controls (lymphocytes; DI = 0.1) A ploidy score between 0.9 and 1.1 was considered diploid, aneuploid 1.1–1.4, triploid 1.4–1.8, tetraploid 1.8–2.2, hypertetraploid >2,2.

### Statistical analysis

Descriptive statistics are calculated with the measures of means, medians and standard deviation for quantitative parameters and counts/percentages for discrete factors. Overall survival is studied with the use of Kaplan Meier method. Survival differences between groups are studied with the use of Log-rank test. A Multivariate Cox regression model was implemented to study the simultaneous effect of parameters on survival after taking into account the parallel effect of remaining factors. Best model selection was based on manual and automated forward techniques. Results of regression analyses are displayed in the form of regression estimates tables. Hazard ratios of outcomes under study are calculated for each parameter estimate as well as 95% confidence intervals. Categorical covariates are compared with a predefined reference category.

All analyses are performed at a significance level of α = 0.05 with the use of the statistical package SPSS 12.0.

## Results

### Patients

Two hundred and twenty six records of patients with histologicaly confirmed pancreatic carcinoma entered the analysis. Their Median age was 62.5 years (range 32–76, median 63.0 and standard deviation: 7.76 years) and the Median PS (Karnofsky) was 70 (range 40–100). Palliative surgery was performed in 52.7% of patients, while 62.9% received chemotherapy for advanced disease. The frequencies of the clinical variables are shown in Table 1.

Despite the fact that authors included in the present study derived from 5 different institutions, it should be stressed-out that all patients during the study period were treated by General Surgeons derived from "Laikon" General Hospital, and all three Medical Oncologists participating in the present study were again affiliated at the same institution (except C.K. who joined "Metaxa" Cancer Hospital after October 2001), and more importantly applied the same therapeutic protocols/algorithms. These treatment algorithms evolved over time in accordance to internationally accepted standards and guidelines with respect to advanced pancreatic cancer. From 1997–2000 a combination of 5FU-Leucovorin (LV)-Epirubicin-Mitomycin (FEMLV) was applied at 1^st^-line followed by single-agent Gemcitabine or the combination of Gemcitabine/CPT11 as 2^nd^-line. From 2001–2003, single-agent Gemcitabine was administered at 1^st^-line and Oxaliplatin-5FU-LV at relapse as 2^nd^-line chemotherapy.

### Survival analysis

Survival data were collected for all patients. Four patients were alive at the end of the study, and their survival time was censored. Based on Kaplan-Meier method, mean survival time was recorded at 38.41 weeks (95% c.i.: 33.17–43.65), median survival: 27.00 weeks (95% c.i.: 23.18–30.82).

### Univariate analysis

In the univariate analysis, all variables were related to the survival outcome at a significance level of alpha = 10%, apart from gender, age and CA19-9 (Table 2).

**Table 2:** Univariate analysis of survival time by categorical variable

Parameter	Log-rank Test value	Degrees of Freedom	P
**Gender**	1,79	1	0,1811
**Age (years)**	0,34	1	0,5586
**PS**	229,07	4	<0.001
**Location**	10,60	2	<0.005
**Grading**	15,22	2	<0.005
**Primary tumor extent**	40,90	3	<0.001
**Distant Metastasis**	38,80	1	<0.001
**Ploidy-group**	93,79	2	<0.001
**Epoetin**	9,16	1	0.003
**Blood Transfusion**	22,64	1	<0.001
**Weight loss**	178,78	3	<0.001
**Pain**	79,81	3	<0.001
**Jaundice**	6,49	1	0.01
**Thrombophlebitis**	16,87	1	<0.001
**Diabetes**	4,36	1	0.04
**Steatorrhoea**	37,96	1	<0.001
**CEA**	37,51	1	0.001
**CA 199**	1,13	1	<0.001
**Amylase**	6,43	1	*0.01*
**CRP**	6,95	2	0.03
**Albumin**	54,78	1	*<0.001*
**Therapy**	161,48	3	*<0.001*

PS: Performance Status; CEA, carcinoembryonic antigen; CA 19-9, cancer antigen 19-9; CRP, C-reactive protein.

### Multivariate analysis

Prognostic factors found to have strongest significance of a relation to survival according to the univariate analysis were entered into the multivariate analysis model first (Table 3). Factors were added and excluded using the change in likelihood between models as inclusion and exclusion criteria. Both manual and forward automated procedures resulted in the same final model, which is described in Table 3.

**Table 3:** Final Cox proportional odds regression model

Variable	B	SE	Wald	p	Hazard ratio	95,0% CI for Exp(B)	
						Lower	Upper
PS(60 vs ≤ 50)	,376	,268	1,966	,161	1,456	,861	2,461
PS(70 vs ≤ 50)	-,501	,319	2,467	,116	,606	,324	1,132
PS(80 vs ≤ 50)	-1,180	,345	11,676	,001	,307	,156	,605
PS(90 vs ≤ 50)	-1,357	,420	10,437	,001	,257	,113	,586

PRIMARY EXTENT(4 vs 1)	,085	,489	,030	,862	1,088	,418	2,836
PRIMARY EXTENT (3 vs 1)	,399	,495	,649	,420	1,491	,564	3,936
PRIMARY EXTENT (2 vs 1)	1,015	,518	3,838	,050	2,758	1,000	7,610

DISTANT METASTASES (yes vs no)	,949	,247	14,730	,000	2,583	1,591	4,194

PLOIDY (3.6+ vs <2.2)	1,605	,341	22,093	,000	4,975	2,548	9,714
PLOIDY (2.2–3.6 vs <2.2)	1,846	,375	24,251	,000	6,333	3,038	13,201

EPOETIN – ANEMIA (yes vs no)	-,441	,184	5,779	,016	,643	,449	,922

WEIGHT LOSS (none vs 10+)	-,536	,379	1,997	,158	,585	,278	1,230
WEIGHT LOSS (1–5% vs 10+)	-1,081	,330	10,719	,001	,339	,178	,648
WEIGHT LOSS (5–10% vs10+)	-1,097	,268	16,756	,000	,334	,197	,565

PAIN (none vs severe)	-,360	,355	1,028	,311	,698	,348	1,399
PAIN (light vs severe)	-,224	,286	,613	,434	,799	,456	1,400
PAIN (moderate vs severe)	-,769	,281	7,486	,006	,463	,267	,804

STEATORIA (yes vs no)	,585	,183	10,185	,001	1,795	1,253	2,572

CEA (>5 vs <= 5)	,345	,161	4,586	,032	1,413	1,030	1,938

THERAPY (CT vs none)	-1,449	,272	28,445	,000	,235	,138	,400
THERAPY (Surgery vs none)	-,416	,254	2,669	,102	,660	,401	1,087
THERAPY(both vs none)	-1,903	,270	49,641	,000	,149	,088	,253

PS, Performance Status; CEA, carcinoembryonic antigen; CT, Chemotherapy.

### Hazard ratios of risk factors

**Figure 3:**
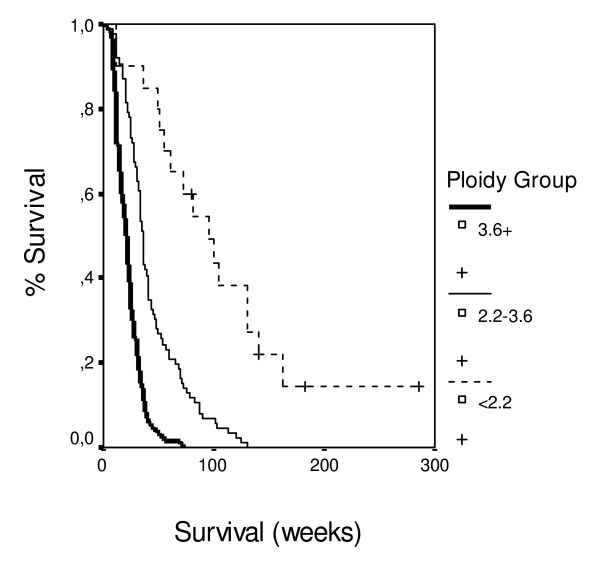
**Survival data according to tumor ploidy**.

**Figure 4:**
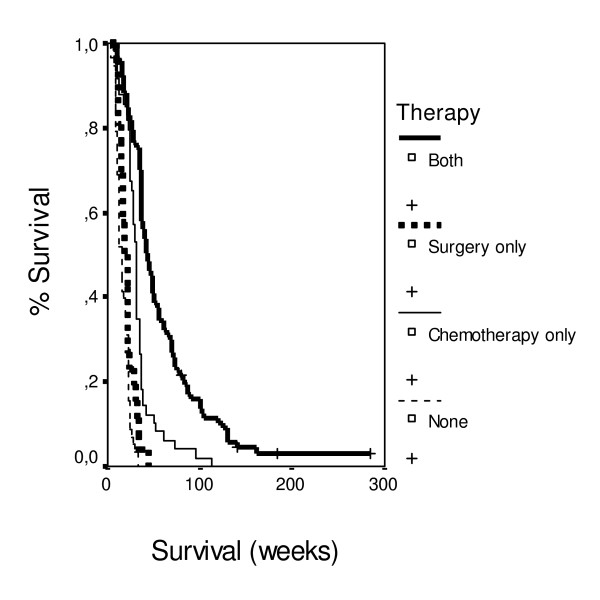
**Survival data according to therapy**.

Patients with PS 80 had 3.0 times lower probability of death in comparison with patients with PS 50, and patients with PS 90 had 3.9 times lower probability of death in comparison with patients with PS 50. Patients with distant metastases in lymph nodes, liver or the abdomen had 2.5 times higher probability of death in comparison with patients without. Patients at local extension of the tumor stage 2 had 2.8 times higher probability of death in comparison with patients at stage 1. Patients with with moderate anaemia under epoetin therapy had 1.5 times lower probability of death in comparison with patients without. Patients with weight loss 1–5% or 5–10% of body weight had 3.0 times lower probability of death in comparison with patients with weight loss > 10%. Patients with steatorrhoea had 1.8 times higher probability of death in comparison with patients without. Patients with CEA > 5 mg/dL had 1.4 times higher probability of death in comparison with patients with CEA < 5 mg/dL. Patients with moderate pain had 2.1 times lower probability of death in comparison with patients with severe pain. Patients with ploidy score 2.2–3.6 had 6.3 times higher probability of death in comparison with patients with ploidy score < 2.2. Patients with ploidy score > 3.6 had 5.0 times higher probability of death in comparison with patients with ploidy score < 2.2 (Figure 3). Patients with only chemotherapy had 4.2 times lower probability of death in comparison with patients without any treatment. Patients with chemotherapy and surgery had 6.7 times lower probability of death in comparison with patients without any treatment (Figure 4).

## Discussion

The majority of patients with pancreatic cancer are not eligible for surgery at presentation because the disease only becomes symptomatic at late stages. Therefore, predictive factors for patients presenting with unresectable, locally recurrent or metastatic disease should be considered when selecting appropriate treatment for each individual patient [6,21-23,25].

The prognostic value of DNA ploidy in pancreatic carcinoma has been studied in the past and results have been disappointing [27]. The first reported evaluation of DNA nuclear pattern in pancreatic cancer was in 1987 by Weger *et al *who used flow cytometry to examine 77 cases of ductal carcinoma [31]. There were no diploid tumor tumors and all patients (n = 16) with triploid neoplasms died within 18 months. In contrast, of 15 patients with near tetraploid tumors, 8 were alive at 70 months after the time of diagnosis. Since then, a number of studies have reported conflicting results in terms of prognostic value of these techniques in pancreatic cancer. Bottger *et al *examined DNA content in 41 cases following resection using image cytometry [32].

Hypotriploid (n = 1), triploid (n = 7), hypertriploid (n = 21), and tetraploid (n = 12) patterns were noted. Tetraploid tumors had a significantly improved survival versus non-tetraploid tumors (p = 0.0037). They found that DNA ploidy was the strongest independent prognostic factor for survival in these patients by multivariate analysis. Yeo *et al *determined DNA content by image cytometry and found that 43% of 119 tumors were diploid and 57% were aneuploid. Patients with diploid tumors had a median survival of 24 months and 5-year survival of 39%, significantly better than the median survival of 11.5 months and 5-year survival of 8% observed in patients with aneuploid tumors; p = 0.0002. In multivariate analysis DNA content was one of the strongest independent predictors of favourable outcome in pancreatic cancer [33]. Porschen *et al *determined DNA content by flow cytometry and found that 29 of 56 (52%) pancreatic ductal adenocarcinomas were diploid, while 27 (48%) were aneuploid. The median survival of those with diploid tumors was 6.9 months as compared to 4.5 months for aneuploid tumors (p = 0.013), but this survival benefit was seen mostly in patients who underwent nonradical surgical intervention. In multivariate analysis, the only factors associated with survival were radicality of surgery and DNA ploidy. The authors concluded that DNA ploidy adds valuable information which is distinct from other clinico-pathological variables [34]. Similar results have been reported by others [35-39]. In contrast, Baisch *et al *using flow cytometry analysis did not find DNA ploidy to be a prognostic factor for survival [40]. They did note that aneuploidy (15%) of their cases was associate with advanced stage and tended to be more common in high grade tumors. Herrera *et al *examined a cohort of 72 patients who underwent radical resection at the Mayo Clinic between 1951 and 1980 [41]. The patients with short (mortality within 12 months) and long term survival (>3 years) were examined. No difference in the DNA nuclear histograms, the fraction of cells in the S phase or DNA index was noted between these two groups. Similarly, in a recently published study, Stoecklein *et al *reported their results of DNA ploidy in conjunction to HER2 amplification and chromosome 17 copy number analysis in patients with pancreatic ductal adenocarcinoma after radical operation (R0 resection). Tumor ploidy levels correlated with prognosis of patients with pancreatic ductal adenocarcinoma, in contrast, to the absence of a prognostic effect on patient outcome regarding *HER2 *gene amplification or p185 (HER2) overexpression [42]. Berczi *et al *also concluded using flow cytometry analysis that DNA ploidy status had no significant effect on survival of patients with carcinoma of the pancreatic head region [43]. To some extend, disparate results in DNA ploidy studies have been ascribed to the differing techniques employed, and the heterogeneity in the nuclear DNA content in pancreatic tumor cells, hence image cytometry has generally been considered superior to flow cytometry as only tumor cells are used for DNA measurement [34]. Subsequently, the clinical utility of DNA ploidy has been limited and its role in staging and treatment is still under investigation [27].

In the present study all factors under investigation were significantly related to survival time except gender, age, and CA-19.9, which is largely in accordance with results previously published by our group and others [28]. We distinguished three groups of factors that influence survival. The first group of factors relates to the presence of disease. Patients with distant metastasis and/or locally extensive disease had a worse prognosis. Patients with increased ploidy score and/or increased levels of the marker CEA, which represent increased tumor burden, were also associated with a worse prognosis. The second group of factors relates to the clinical characteristics of the patient and the immune response such as poor PS at the time of diagnosis, weight loss, severity of pain, steatorrhoea, and anaemia under epoetin-A therapy. Each of these factors was related to a negative influence on survival. The third group of factors relates to therapeutic modalities: patients given only supportive care had the worst survival in comparison to those who underwent chemotherapy and better with the combination of chemotherapy and surgery.

According to the significance of the examined factor, survival was improved mainly by the combination of surgery and chemotherapy, and by a low DNA ploidy score. Secondary factors are the performance status (performance status and weight loss) and expansion of cancer (local extension and distant metastases). Considerable factors are pain, anemia, steatorrhoea, and CEA value.

The present study represents a comprehensive analysis regarding the prognostic significance of microscopically assessed DNA ploidy in the context of additional patient- and treatment-related known prognostic factors in a large cohort of advanced inoperable ductal pancreatic adenocarcinoma. Our analysis demonstrated that DNA ploidy, together with chemotherapy or surgery or surgery plus chemotherapy carry the most significant independent effect on outcome of advanced pancreatic adenocarcinoma.

## Conclusion

In conclusion, we assessed factors that are related to the outcome of patients with advanced, unresectable ductal pancreatic adenocarcinoma. The determination of DNA content of pancreatic cancer cells provides additional prognostic information and should be considered along with other established prognostic factors for the development of effective multidisciplinary treatment strategies and the design of future clinical trials. In terms of treatment modality our study reinforces the notion that the main factor, which may affect survival in patients with pancreatic cancer is therapy, and so all patients, unless contraindicated, should be offered palliative surgery and chemotherapy, or chemotherapy alone if the tumor is considered unresectable.

## Competing interests

The authors declare that they have no competing interests.

## Authors' contributions

NT conceived of the study, and participated in its design and coordination and helped to draft the manuscript. NK, AL participated in study design and laboratory work. KT participated in study design, collection of the data and laboratory work. IDX and CK were involved in data interpretation, critical review and helped to draft the manuscript. NP, AD, AP and JS participated in the collection of clinical data (clinical oncology). GA: performed part of laboratory work and was involved in the collection of laboratory data. EF, EA, HT and GK participated in the collection of clinical data (surgical oncology). EP coordinated the collection of clinical data (surgical oncology). ESP coordinated the collection of laboratory data. All authors read and approved the final manuscript.

## Pre-publication history

The pre-publication history for this paper can be accessed here:

http://www.biomedcentral.com/1471-2407/9/264/prepub
